# A new era of cancer immunotherapy: vaccines and miRNAs

**DOI:** 10.1007/s00262-025-04011-5

**Published:** 2025-04-01

**Authors:** Gitika Sareen, Maneesh Mohan, Ashi Mannan, Kamal Dua, Thakur Gurjeet Singh

**Affiliations:** 1 Chitkara College of Pharmacy, Chitkara University, Rajpura, Punjab 140401 India; 2 Discipline of Pharmacy, Graduate School of Health, University of Technology Sydney, Ultimo, NSW 2007 Australia; 3 Faculty of Health, Australian Research Centre in Complementary and Integrative Medicine, University of Technology Sydney, Ultimo, NSW 2007 Australia

**Keywords:** Cancer vaccines, Immunotherapy, MicroRNAs (miRNAs), Combination therapy, Tumor immunogenicity

## Abstract

Cancer immunotherapy has transformed the treatment landscape, introducing new strategies to fight various types of cancer. This review examines the important role of vaccines in cancer therapy, focusing on recent advancements such as dendritic cell vaccines, mRNA vaccines, and viral vector-based approaches. The relationship between cancer and the immune system highlights the importance of vaccines as therapeutic tools. The discussion covers tumor cell and dendritic cell vaccines, protein/peptide vaccines, and nucleic acid vaccines (including DNA, RNA, or viral vector-based), with a focus on their effectiveness and underlying mechanisms. Combination therapies that pair vaccines with immune checkpoint inhibitors, TIL therapy, and TCR/CAR-T cell therapy show promising potential, boosting antitumor responses. Additionally, the review explores the regulatory functions of microRNAs (miRNAs) in cancer development and suppression, featuring miR-21, miR-155, the let-7 family, and the miR-200 family, among others. These miRNAs influence various pathways, such as PI3K/AKT, NF-κB, and EMT regulation, providing insights into biomarker-driven therapeutic strategies. Overall, this work offers a thorough overview of vaccines in oncology and the integrative role of miRNAs, setting the stage for the next generation of cancer immunotherapies.

## Introduction

Cancer represents a diverse group of diseases with uncontrolled growth and spread of neoplastic cells, which escape normal cellular controls, invade nearby tissues, and can metastasize to distant organs. Impairing their regular operations, these cells can infiltrate healthy tissues and potentially metastasize to other body areas. A noticeable reduction in DNA methylation, which lowers the overall quantity of 5-methyl cytosine by 5–6%, is one of the distinct features of cancer cells, contributing to the widespread health issue [1]. This category of disease is the second leading cause of mortality worldwide, underscoring its extensive effect on humans [2]. The incidence of cancer differs geographically and is impacted by a wide range of factors, such as exposure to carcinogens, lifestyle choices, age, gender, and genetics. Data from the 1991 Indian census indicate that approximately 609,000 instances of cancer have been reported. The nineteenth century saw the first cancer diagnoses and the twentieth century saw a rise in the disease’s incidence. The International Agency for Research on Cancer projects that by 2035, cancer cases in India will rise to almost 1.7 million, up from 1 million cases in 2012. They also estimate that the death rate from cancer will rise from 680,000 to 1–2 million during the same period [3]. So in 2018, the anticipated incidence of cancer in India was around 1.15 million cases and is expected to nearly quadruple by 2040 due to demographic changes alone [4].

Moreover, infectious microorganisms substantially influence the initiation and risk of cancer. These agents comprise many pathogens, such as bacteria, viruses, and parasites. Common viral aetiologies linked to cancer development include hepatitis B/C virus (HBV/HCV), aflatoxin exposure, *Helicobacter pylori* (H. pylori) infection, human papillomavirus (HPV), and Epstein–Barr virus. These viruses are associated with an elevated risk of acquiring specific cancer forms. These infections are also significant risk factors for particular cancer types. For example, they have been connected to gastric, liver, cervical, and nasopharyngeal cancers in that order [5]. Furthermore, a recent estimate placed the primary cause of approximately 1,400,000 cancer cases annually attributable to viral infections, accounting for around 10% of the global cancer burden. Oral cavity and laryngeal malignancies have an attributable fraction of 4%, but cervical carcinomas have a nearly 100% attributable proportion [6]. Moreover, in males, the prostate, lung and bronchus, colon and rectum, and urinary bladder have the highest prevalence of cancer kinds. In females, the breast, lung and bronchus, colon and rectum, uterine corpus, and thyroid exhibit the most significant cancer incidence rates. Prostate cancer is a major contributor to cancer in males, whereas breast cancer significantly affects cancer incidence in women. In order, the most common cancers in children are blood, brain, and lymph nodes [2].

A condition is known as cancer that leads to different alterations in the well-being of cells and tissues, eventually leading to the development of malignant tumors. In cancer, the biological outcome is known as neoplasia, which refers to abnormal cell growth. For many individuals with cancer, the primary cause of illness and mortality is the invasion of tumor cells into nearby tissues and their subsequent spread, also known as metastasis, to distant organs [7]. Even with better methods to identify and address pain associated with cancer treatment, it continues to be a frequent and persistent symptom, affecting as many as 55% of individuals following cancer treatment [8]. A tumor represents an unusual proliferation of cells that can infiltrate surrounding tissues, organs, and distant regions within the body. When cells multiply abnormally and start invading nearby tissues, organs, or even distant body parts, we refer to it as a tumor. These proliferating cells are not always cancerous and have no useful purpose. There exist benign tumors characterized by their limited tendency to metastasize or disseminate to other body regions. Various forms of cancer pose a threat to human health, yet the insidious nature of cancer cells during their initial growth phases often results in the unrecognized onset of these problems [9]. To maintain their metabolic demands and ensure survival, cancer cells have evolved systems to regulate the entry and efflux of Ca2 + within their mitochondria. Thus, one possible treatment strategy for cancer patients could be to target the mitochondrial Ca2 + signaling implicated in the bioenergetic and apoptotic pathways [10]. The pathophysiology of cancer refers to the process by which genetic mutations or alterations in a normal cell’s DNA cause the cancer to start. The altered cell, often called a cancer cell, cannot control its division and growth [11]. Next, a tumor begins to form, and as it enlarges, it needs blood flow to get nutrition and oxygen. Metastasis is a critical phase in cancer progression that often leads to mortality [12]. The immune system, which usually detects and eradicates abnormal cells, might be deceived by cancer cells. Cancer can have several systemic impacts on the body as it advances, including immune system suppression, exhaustion, and weight loss. Genetic alterations, growth trends, and treatment responses can vary throughout cancer.

Modifying your diet by increasing the consumption of fruits, vegetables, and whole grains while decreasing red and processed meat, engaging in regular physical activity, sustaining a healthy weight, and abstaining from alcohol and tobacco is thought to prevent 30 to 50 percent of cancer cases [13]. In addition to non-pharmacological approaches, surgical techniques, chemotherapy, and radiation therapy have been successful cancer treatments during the past century. When employed singly or in combination, these therapeutic approaches can substantially impact tumor progression and perhaps lead to cures [14]. The use of other medications that may raise the incidence of anthracycline-induced cardiotoxicity raises the risk of it. Specifically, trastuzumab disrupts the pathways that myocytes use to survive, which is essential to preventing the harmful effects of anthracyclines despite being a very effective treatment for breast cancer [15]. Anticancer drugs can cause various toxicities, including cardiotoxicity (doxorubicin, epirubicin), hepatotoxicity (crizotinib, afatinib, dasatinib), neurotoxicity (cisplatin, paclitaxel, docetaxel, bortezomib, thalidomide) [16], and pulmonary toxicity (paclitaxel, docetaxel) [17]. Other drugs associated with cardiotoxicity include oxaliplatin, carboplatin, cisplatin, ifosfamide, mitomycin C, bleomycin, methotrexate, vinca alkaloids, bendamustine, nitrosoureas, melphalan, gemcitabine, capecitabine, pemetrexed, and irinotecan. It is important to note that many of these drugs can cause multiple toxicities, and they can also cause nephrotoxicity [18]. Higher levels of financial toxicities were linked to poorer quality of life as well as patient outcomes, including lack of insurance and lower socioeconomic position [19]. Immunotherapy is a new method that bypasses the limitations by leveraging the body’s immune system to recognize and destroy cancer cells. Immunotherapy provokes the immune system to provide it with more specific and potentially long-term protection against cancer than conventional therapy, which attacks tumors themselves. Immune checkpoint inhibitors, monoclonal antibodies, cancer vaccines, and adoptive cell therapies—e.g., CAR-T cell therapy—are all part of this innovative approach and have shown much promise in enhancing patient outcomes.

Vaccines are another method of treating cancer. Also, cancer vaccines with therapeutic purposes have the potential to halt the progression of advanced cancers and relapsed tumors that do not respond to traditional treatments like radiation therapy, chemotherapy, and surgery. Natural bioactive substances can be added to conventional chemotherapeutic medications to increase their anticancer benefits and lessen their adverse effects [20]. In rare circumstances, including bioactive substances may help cancer cells overcome their resistance to chemotherapy or radiation [21]. Therapeutic cancer vaccines aim to prevent non-specific or unpleasant reactions, build persistent antitumor memory, remove minimal residual illness, and drive tumor regression [20]. Besides creating vaccines, scientists have also started investigating microRNAs as a novel cancer prevention and treatment approach. MiRNAs, also known as microRNAs, are a special kind of non-coding RNAs. They are approximately 19–24 long nucleotides and are crucial in regulating gene expression post-transcription, affecting numerous biological processes [22]. Because of their many functions and involvement in clinical states, miRNAs have great potential as therapeutic agents, particularly for diseases that do not have a clear genetic cause.

## Immune responses in cancer

The complex interplay between cancer cells and the immune system underlies significant progress in immunotherapeutic approaches. Tumors often exploit tissue-resident immune cells, endothelial cells, fibroblasts, and neurons to their advantage, utilizing their contribution to promoting tumor growth. Immune cells like tumor-associated neutrophils (TANs), regulatory T cells (Tregs), tumor-associated macrophages (TAMs), and myeloid-derived suppressor cells (MDSC) play a role in facilitating tumor growth [23].

These immune cells are modulated through chemokines, cytokines, and effector molecules like collagen, matrix metalloproteinases (MMPs), laminin, TGF-β, CXCL2, and cancer-associated fibroblasts (CAFs). Their activation contributes significantly to tumor development, extracellular matrix remodeling, and degradation [24]. This dynamic interaction between immune cells and tumors causes metabolic competition in the ecosystem surrounding the tumor, which reduces the number of nutrients available and causes microenvironmental acidosis, which impairs the ability of immune cells to function [25]. Recent studies reveal that cancer cells can inhibit the body’s immune response to tumors by diminishing the metabolic fitness of immune cells that infiltrate tumors or by competing for and utilizing vital resources. The innate immune system comprises many immune cells, including eosinophils, neutrophils, natural killer cells, basophils, monocytes, and macrophages, responsible for innate immunity against pathogens to maintain the host’s homeostasis [26].

The connection between the immune system and cancer is comprised of three stages. In the initial stage, innate and adaptive immune cells eradicate tumor cells (elimination phase). The equilibrium phase ensues, during which the immune system cannot eliminate the tumor, but its growth can be managed. Through various means, tumor cells escape immune control during the third phase, which leads to a condition that may be clinically detected [27]. Immunologically, tumors can be classified as either inflammatory or non-inflammatory based on the extent of immune cell infiltration and their activation level. Immunotherapies are typically more effective for inflammatory cancers than non-inflammatory ones. However, immune cells are observed close to the invasive borders of tumors in the immune-excluded type [28]. Regulatory T cells (Tregs) can impede the immune system’s capacity to identify cancer in healthy individuals and diminish its efficacy in combating tumors in affected hosts. Consequently, Tregs enhance the capacity of tumor cells to circumvent the immune system, resulting in expedited tumor development and dissemination across multiple cancer types. As a result, Tregs are regarded as an essential therapeutic target for immunotherapy against cancer. Numerous cancer types exhibit abundant Tregs in the tumor microenvironment (TME), including in inflammatory and non-inflammatory cancers [28, 29].

Cancer immune surveillance is a crucial host defense mechanism for preventing cancer and preserving cellular homeostasis. The process is categorized into three principal stages: escape, wherein immune-resistant cancer cell clones proliferate and generate tumors; equilibrium, during which the immune system applies selective pressure to eradicate the most immunogenic cancer cell clones; and elimination, wherein the newly transformed cells are identified and eradicated by cytotoxic CD8 + lymphocytes, natural killer (NK) cells, and other immune cells [30]. One of the defining characteristics of carcinogenesis is cancer immune evasion. Cancer cells inhibit the immune system by complexly modulating immune-related pathways at the transcriptional, translational, and post-translational levels. Specifically, it has been demonstrated that DNA methyltransferases 1 (DNMT1), 3A, and 3B are significant de novo and maintenance methyltransferases in cancer [31]. Various immune invasion strategies can impact MHC (major histocompatibility test) class I/peptide presentation and the differentiation, production, migration, survival, and proliferation of particular cytotoxic T cell clones, which can select cancer cells during tumor progression. We created rat CHO cell lines that either overexpress proteins known to possess the capacity to evade our immune response or that have an immunosuppressive agent added to the culture media. These cell lines express human EpCAM (epithelial cell adhesion molecule) as the surface target antigen. The efficacy of cytotoxic T cells was assessed by evaluating the magnitude and effectiveness of redirected CHO-EpCAM target cell lysis and the stimulation of T cell proliferation in response to AMG 110. We examined chemicals frequently generated by cancer cells interacting with negative regulatory surface receptors on cytotoxic T lymphocytes, specifically PD-L1, adenosine, IL-10, and TGF-β. Tumor cells generate PD-L1, and monoclonal antibody (mAb) treatment inhibits T cell attachment to its inhibitory receptor, PD-1 [32]. It has been seen that T cells can kill cancerous cells and that the human immune system can eradicate them by employing T cells to carry out acquired immune responses. These findings imply that T cells may be logically engineered to regulate the formation of tumors [33]. Still, the immune system’s ability to eradicate tumor cells is a complicated process that depends on several factors. Tumor-associated antigens are initially discharged from necrotic tumor cells into the adjacent tissue and subsequently captured by antigen-presenting cells (APCs). Antigen-presenting cells (APCs) process major histocompatibility complex (MHC) and antigens, presenting them on the cell surface and transferring them to lymphoid organs. Primitive T cells in lymphoid organs recognize specific peptide major histocompatibility complexes via the T cell receptor (TCR), initiating the priming and activation of effector T cells [34].

T cell-based immunotherapy is currently recognized as a crucial component of cancer treatment [34]. Immunotherapy is a substantial breakthrough in cancer treatment that has fundamentally transformed the field of oncology. The objective is to enhance the body’s immunity against malignant cells. Given that immune cells provide the biological basis of immunotherapy, it is crucial to understand the immunological infiltrates within the tumor microenvironment (TME) to improve response rates and develop innovative therapeutic strategies for cancer treatment. Extensive research has been conducted on T cells; however, other immune cells from both the innate and adaptive immune systems, including macrophages, natural killer (NK) cells, dendritic cells (DCs), and B lymphocytes, have also been shown to contribute to tumor progression and responses to immunotherapy [35].

In the fight against cancer, immunotherapy has revolutionized the field. Fighting tumor cells depends on strengthening the patient’s immune system. Since the FDA-approved chimeric antigen receptor (CAR) T cell therapy, immunotherapy has also been successful in adoptive cell therapies. Other promising approaches include tumor-infiltrating lymphocytes (TILs) and T cell receptor (TCR)—engineered cells, which are being tested in multiple advanced clinical trials for various cancers [36].

## Vaccines as cancer immunotherapy

A vaccine’s goal is to either prevent or lessen the severity of infectious diseases that can be fatal (prophylactic vaccines). Vaccines, both therapeutic and preventive, serve as exemplary approaches to cancer immunotherapy. Since infectious viruses bring on some malignancies, vaccines against viruses can help prevent cancer formation in the first place. To prevent hepatitis B virus (HBV)-related hepatocarcinoma and human papillomavirus (HPV)-related malignancies, the Food and Drug Administration (FDA) has approved two types of prophylactic cancer vaccines [37]. Vaccines have also been used as therapeutic approaches, triggering the immune system to launch cytotoxic T cells to fight against malignancies and infected cells. The cancer models where the etiological oncogenic agents are foreign viruses, such as human papillomavirus-associated malignancies, have shown the most tremendous success with DNA vaccines [38].

Cancer vaccines can elicit immunological memory that offers long-term protection against tumor recurrence, promote targeted destruction of tumor cells with minimum harm to healthy cells, and trigger particular anticancer immune responses. As potential cancer vaccine candidates, CT antigens have been studied [39]. Tumor-specific antigens are used in cancer vaccines to initiate T cell-mediated immune responses against tumors. The discovery of MZ2-E and MZ2-D, two melanoma-derived antigens produced by the MAGE (melanoma-associated antigen) gene family, which cytotoxic T lymphocytes could detect to initiate anticancer immune responses, led to pivotal investigations [40]. The most proficient antigen-presenting cells (APCs) are DCs, essential for inducing anticancer immunity. This procedure involves reinfusing isolated dendritic cells that have been pulsed with tumor antigens or tumor cell lysates and activated ex vivo using a designated maturation cocktail. The advancement of GVAX, a cancer vaccine composed of genetically modified autologous tumor cells that secrete granulocyte–macrophage colony-stimulating factor, demonstrated potential in augmenting tumor-specific immune responses across multiple cancer types [39]. Prophylactic vaccines against the human papillomavirus (HPV) are founded on the appealing and straightforward idea that malignancies caused by oncogenic HPV infections can be avoided through antibody-mediated prevention of HPV infections. Every year, HPV vaccines could potentially save over 500,000 cases of oral, anogenital, and cervical cancer globally. Immunotherapy aimed at the cancer-associated viral early proteins (HPV16 E6 and HPV16 E7) is anticipated to establish the basis for cancer-specific immunotherapy, as HPV-associated tumors persist in expressing viral proteins and presenting them to the host immune system. The MUC1 (Mucin1) vaccine is both safe and highly immunogenic. It can impede the development of new adenomas in persons with premalignant colonic adenomas, hence reducing the risk of colon cancer [41].

Cancer vaccines have been powerful tools for prophylactic as well as therapeutic immunotherapy that can be used to prevent or reduce the severity of malignancies caused by infectious agents and stimulate immune responses against cancer [42]. FDA-approved prophylactic vaccines include those for hepatitis B virus (HBV) to avert hepatocellular carcinoma and human papillomavirus (HPV) to reduce the incidence of cervical and anogenital cancers. Therapeutic cancer vaccines, including DNA vaccines, have been effective against cancers caused by HPV through the induction of cytotoxic T cell responses [43]. Cancer vaccines stimulate immunological memory, providing long-term immunity against the recurrence of tumors and minimizing the destruction of normal cells. Antigens, such as melanoma-associated antigens (MAGE), have been critical in the evolution of cancer vaccines, triggering T cell-mediated immune responses [44]. Dendritic cell (DC)-based vaccines, like GVAX, have shown promise in augmenting tumor-specific immunity. Recent developments are mRNA-based cancer vaccines such as BNT162b2, which utilize lipid nanoparticle technology to elicit strong immune responses, and CRISPR-edited CAR-T cells, which increase the specificity of T cell-mediated tumor targeting [45]. These technologies are transforming cancer immunotherapy by providing highly individualized and efficient treatment approaches. Cancer vaccines and microRNAs (miRNAs) complement each other in cancer immunotherapy by augmenting immune response and regulating gene expression in the tumor microenvironment. Vaccines trigger the immune system to identify and target cancer cells by presenting tumor-associated antigens, activating cytotoxic T cells and long-lasting immunologic memory. In contrast, miRNAs control critical immune-related pathways by regulating antigen presentation, cytokine secretion, and immune cell differentiation. Some miRNAs can improve vaccine effectiveness by increasing immune activation, whereas others can be involved in immune evasion by repressing antitumor immunity. The integration of cancer vaccines with miRNA therapy has the potential to overcome immune resistance, enhance tumor targeting, and increase the persistence of immunotherapy responses.

### Cell vaccines (tumor cell vaccines or dendritic cell (DC) vaccines)

Dendritic cells (DCs) are essential antigen-presenting cells in the immune system derived from hematopoietic stem cells in the bone marrow. They play a pivotal role in orchestrating innate and adaptive immune responses to pathogens, including tumor antigens [46]. Nevertheless, cancer disrupts this process, resulting in a reduction in the number and functionality of DCs. This dysfunction underscores the potential of DCs as a therapeutic target. DC vaccines are promising for augmenting the immune system’s capacity to combat cancer. DC-based therapy has demonstrated the ability to augment T cell priming and modify the tumor microenvironment, thereby augmenting systemic host immune responses and achieving long-term antitumor responses [47]. DC vaccines are intended to enhance the immunogenicity of tumor-associated antigens. In this method, autologous dendritic cells are cultured with necrotic lung cancer cells to facilitate the processing of the tumor antigens. Subsequently, the antigen-loaded dendritic cells are administered intradermally. Therapeutic cancer vaccines are designed to increase tumor-specific T cell immunity. A primary goal of therapeutic cancer vaccines is to promote tumor regression by inducing antigen-specific T cells in vivo. SNP-IV was able to control the growth of established tumors; this was associated with the generation of stem-like CD8 + T cells capable of replenishing effector cells upon treatment with checkpoint inhibitors such as anti-PD-L1 [37, 48]. Dendritic cells (DC) are well known as the optimal antigen-presenting cell (APC) for priming T cell responses. DC requires minimal amounts of antigens to stimulate T cell proliferation. They are also shown to be superior stimulators of T cells, so 100-fold more macrophages and B-cells are needed to induce a proliferative MLR (mixed lymphocyte reaction) response. The recognition mechanisms that DC uses to recognize endogenous and non-endogenous signals are also another strategy currently utilized to generate more effective DC vaccines against cancer by taking advantage of highly immunogenic signals resulting from cell death [49]. cDCs (conventional dendritic cells) can be further subdivided into type 1 (cDC1) or type 2 (cDC2) lineages: cDC1s excel at cross-presentation for priming of CD8 + T cells, whereas cDC2s are specialized at priming CD4 + T cells [48].

**Fig. 1:**
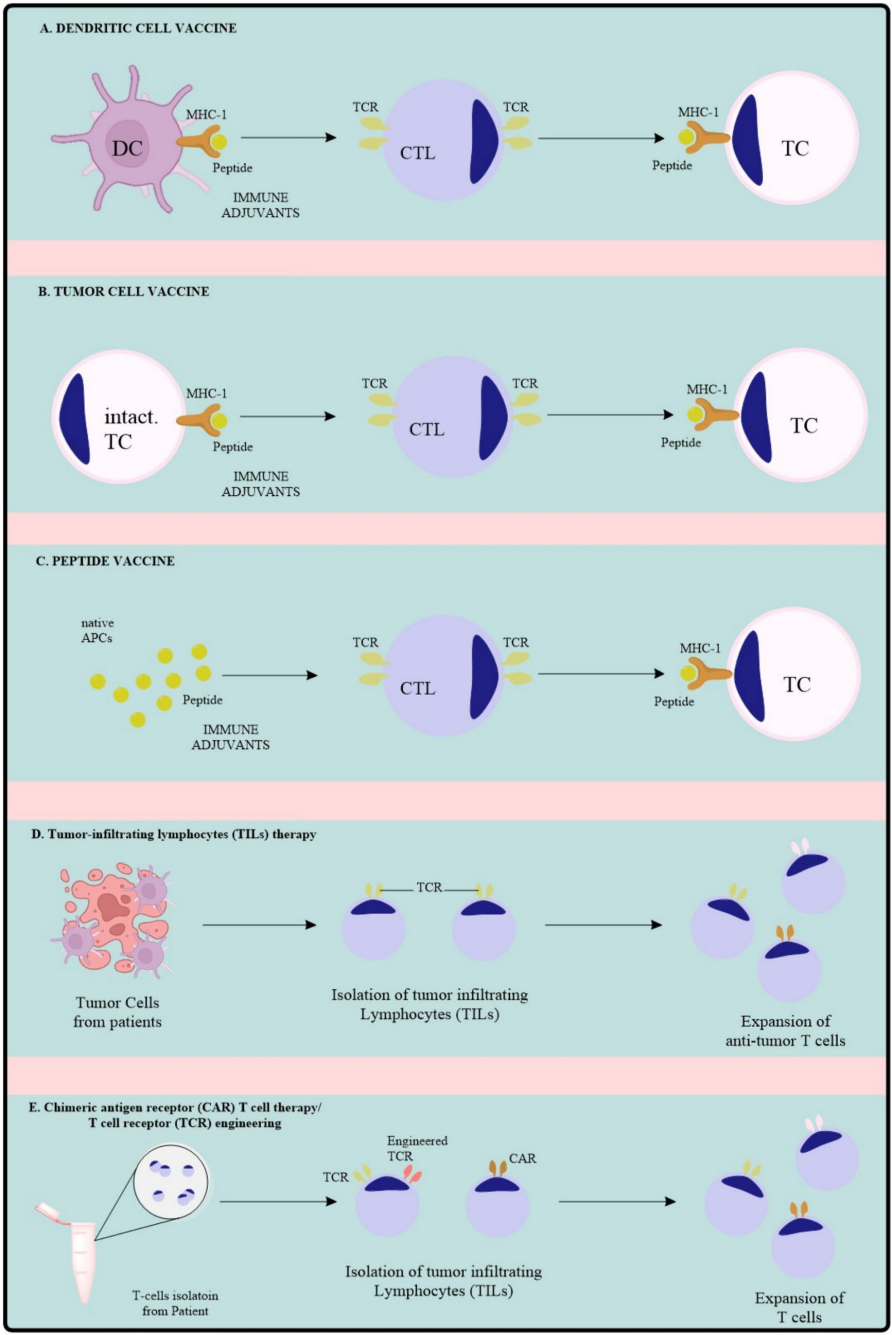
Types of vaccines and therapies

Spileucel-T, a therapeutic cancer vaccine consisting of autologous peripheral blood mononuclear cells (DCs) fused with granulocyte–macrophage colony-stimulating factor (GM-CSF), which serves as an immune cell activator and is loaded with the prostatic acid phosphatase antigen, was the inaugural vaccine approved by the US Food and Drug Administration. These attributes underscore the importance of extensive activation of CTLs and T helper cells through two mechanisms: selecting appropriate antigens that activate both T cell populations and systematically designing vaccines for the precise delivery of tumor antigens to activated dendritic cells, enabling the loading of epitopes from exogenous tumor antigens onto MHC class I (via the cross-presentation pathway) and MHC class II molecules to stimulate CTLs and T helper cells effectively [50]. To achieve these requirements, therapeutic cancer vaccine techniques have evolved over the past ten years, combining better antigen selection, immunogenicity, and structural design [47]. Murine models have provided critical insights into the antitumor mechanisms of DC vaccines, specifically the stimulation of cytotoxic T cells and the diminishment of neoplastic tissue. These vaccines hold significant potential for cancer treatment. Various murine models provide supporting evidence for DC vaccines [51]. One study illustrates that successful treatment of DC infused with tumor peptides is contingent upon T cells, B7 costimulation, and Th1 cytokines. In models such as B16 melanoma and MBT-2 bladder tumors, the vaccines with DCs laden with unfractionated tumor lysates significantly extended survival and reduced lung metastases. These results emphasize the capacity of DC vaccines to induce tumor-specific CTL responses without necessitating the identification of specific antigens in each instance [52]. Although showing promising preclinical results, dendritic cell (DC) vaccines have shown only limited efficacy in the clinic and limited long-term survival advantage. One significant hindrance is the immunosuppressive tumor microenvironment, which suppresses the immune activation and hinders the vaccine from functioning effectively. Another issue is the manufacturing process, which is complicated and expensive, involving individualized ex vivo preparation, thus posing difficulty for large-scale production. Even if successful, the immune response is usually fleeting, requiring booster shots or combination regimens to maintain efficacy. In addition, tumor antigen heterogeneity permits tumor cells to avoid immune recognition, reducing vaccine efficacy. Finally, ambiguities about the ideal dose, timing, and delivery route add to the challenges of implementing DC-based treatments on a large scale (Fig. 1).

### Protein/Peptide vaccines

Antibodies exhibit a higher affinity and a prolonged half-life than peptides. In contrast, antibodies are less effective than peptides regarding cell internalization and tissue penetration. Despite the merits and demerits of peptides, they have been utilized as tumor-targeting ligands to deliver carriers (including nanoparticles, extracellular vesicles, and cells) and payloads (such as cytotoxic peptides and radioisotopes) to tumors [53]. Furthermore, linkers have been employed to conjugate tumor-homing peptides with cargoes, including small-molecule or chemotherapeutic medications, to produce peptide–drug conjugates [53]. Moreover, peptides specifically interacted with cell surface receptors and proteins, such as immunological checkpoints, receptor kinases, and hormone receptors, suppressing their biological function or serving as hormone analogs. Furthermore, internalized peptides within cells associate with intracellular proteins, disrupting protein–protein interactions. Thus, peptides demonstrate considerable promise as multifunctional agents in cancer treatment [53]. Protein/peptide vaccines are a form of immunotherapy that induces an immune response against specific proteins or peptides linked to diseases, with a particular emphasis on cancer. These vaccines comprise complete proteins that are derived from tumor cells or pathogens.

Peptide vaccines exhibit significant promise in cancer immunotherapy by targeting tumor antigens and stimulating the patient’s immune system to elicit a particular response against cancer cells. Peptide vaccines have numerous advantages compared to other vaccine types, such as their selectivity for neoplastic cells, potential for enduring immunological memory, ease of production and scalability, and low toxicity. Upon administration to patients, Peptide vaccines are digested and presented by antigen-presenting cells (APCs), such as macrophages and dendritic cells (DCs), activating T cells to initiate an immune response against cancer cells [54]. Peptide-based hydrogels are supramolecular materials that self-assemble into diverse nanostructures via several interacting forces (e.g., hydrogen bonding and hydrophobic interactions) and react to microenvironmental stimuli (e.g., pH and temperature). Immunotherapies aim to utilize the body’s immune system to safeguard against and address numerous diseases, including cancer proactively [55]. Furthermore, several studies using HPV16 mE6Δ/mE7/TBhsp70Δ fusion protein in a C57BL/6 mouse model demonstrate the promising approach, effectively preventing and treating HPV-associated cancers and metastases. It has been demonstrated that vaccinating inbred rodents with these peptides produces a protective immune response against tumors [56]. The mechanisms involve the peptide presenting the associated tumor antigen to cytotoxic T cells, which mount a target against the cancer cells. Developing a peptide vaccine targeting HER1, HER2, and VEGF is also highly promising. Peptide cancer vaccines are encumbered with a number of challenges that hamper their clinical efficacy. Their low immunogenicity is one significant challenge, wherein they need adjuvants or combination therapies to induce a potent immune response. Moreover, HLA restriction limits their efficacy in the population at large, as they are tailored to attack specific human leukocyte antigens (HLAs). They also have a short half-life, making repeated doses a necessity to maintain efficacy. In addition, antigen escape variability enables cancer cells to mutate and become immune evasive, reducing vaccine efficacy with time. Although peptide vaccines have demonstrated potential in virus-induced cancer prevention, including HPV-related cancers, numerous others have failed late-stage clinical trials because they failed to stimulate the immune system adequately, making optimization necessary (Fig. 1).

### Nucleic acid vaccines (DNA, RNA, or viral vector)

Nucleic acid vaccines are a type of vaccines that elicits an immune response through the use of genetic material, including DNA, RNA, or viral vectors. These vaccines possess the capacity to be both commercially viable and efficacious. Nucleic acid-based vaccines, comprising DNA (in the form of plasmids) and RNA (as messenger RNA [mRNA]), demonstrate considerable promise for the management of triple-negative breast cancer (TNBC) [57]. Nucleic acid vaccines (NAVs) have lately been explored as a therapeutic approach for cancer. DNA and mRNA vaccines convey genetic information encoding tumor antigens (TAs) to the host, hence eliciting immune responses against cancer cells expressing the TAs [58]. Notwithstanding the convenience, safety, and straightforwardness of producing NAVs, they have not yet been regarded as feasible substitutes for peptide vaccines. This method faces multiple challenges, such as the immunosuppressive characteristics of cancer, inadequate immunogenicity, and the identification of suitable tumor antigens [59]. Therapeutic DNA cancer vaccines are seen as a promising approach to stimulate the immune system against cancer. DNA vaccines may be optimized through two distinct ways to augment the immune response elicited by the vaccines and improve treatment efficacy. The initial approach involves enhancing immunogenicity by the selection and optimization of the most effective antigen(s) included into the plasmid DNA [60]. The second technique involves integrating DNA vaccines with additional complementary medicines to enhance their efficacy by mitigating immunosuppression within the tumor microenvironment or augmenting the activity and quantity of immune cells. Several commercially available immunotherapeutic therapies include anti-CTLA-4, anti-PD-1, anti-PD-L1, and CAR-T cells targeting acute lymphoblastic leukemia and B-cell lymphoma, among others [61]. mRNA vaccines represent a compelling and strong immunotherapeutic platform for cancer due to their high efficacy, specificity, adaptability, quick and scalable development capabilities, cost-effective manufacturing potential, and safety profile. The immunostimulant mRNA vaccine TriMix, which encodes CD70, CD40L, and a constitutively active version of TLR4, elicited robust CD8 + T cell responses in patients with stage III or IV melanoma, demonstrating favorable tumor response rates in a phase II clinical trial [62]. The safety and antitumor efficiency of viral-based vaccines in preclinical models have prompted clinical trials to assess the immunological and clinical effectiveness of this therapy approach. Viral vectors comprise derivatives of vaccinia virus from the orthopoxvirus genus and members of the avipoxvirus genus, including fowlpox and canarypox (ALVAC). Poxviral vectors have a broad host range, stable recombinants, precise replication, and effective post-translational processing of added genes. The intracellular expression of the transgene(s) facilitates the processing of the tumor antigen via both class I and II MHC pathways, resulting in the activation of CD4 + and CD8 + T cells [63]. Several studies show that nucleic acid vaccines have a promising role in preclinical studies. The robust antitumor immunity observed in CT26 and B16F10 murine models, with sustained T cell responses against tumor-specific neoepitopes, is particularly encouraging [64]. This approach of DNA vaccines targeting NMM antigens effectively induced cellular immunity and inhibited tumor growth. These studies highlight the potential of DNA vaccines for personalized therapy design. Another study on nucleic acid vaccines demonstrated that a the DNA vaccines encoding NMM (NY-ESO-1/MAGE-A3/MUC1) target antigens and immune-related components successfully elicited cellular immune responses and suppressed tumor growth in C57BL/6 mice, providing a novel approach for tumor vaccine design and laying a foundation for clinical application [65]. The pNMM vaccine showed significant immunogenicity and efficacy in prolonging survival in mice with B16-NMM + tumors. A study on globin-stabilized mRNA vaccines in mice shows successful translation and modulation of the immune response, presenting a versatile and safe alternative to DNA vaccines for various diseases, including antiviral, antibacterial, and antitumor applications [66]. The use of naked β-globin UTR-stabilized mRNA encoding β-galactosidase was evaluated, revealing detectable translation in vivo and a Th2-type immune response that can be augmented and redirected to a Th1-type response by recombinant granulocyte/macrophage colony-stimulating factor [67]. This study demonstrates that plasmid DNA-based neoepitope vaccines induce robust antitumor immunity in CT26 and B16F10 murine cancer models, with sustained neoepitope-specific T cell responses, highlighting their potential as personalized cancer immunotherapies. A separate study assesses the effectiveness of a xenogeneic EGFR DNA vaccine delivered via various methods and formulations in a murine lung tumor model. The gene gun delivery of the non-coating DNA vaccine elicited the most robust cytotoxic T cell activity and optimal anticancer effects. CD8( +) T cells were crucial for antitumor immunity, suggesting this method as a viable strategy for treating EGFR-positive lung tumors [68]. Nucleic acid vaccines, such as DNA and mRNA-based strategies, encounter a number of key challenges that limit their general success in cancer treatment. A significant challenge is low transfection efficiency, as DNA vaccines are unable to enter cells efficiently, leading to poor immune responses. Another significant challenge is mRNA instability, since these molecules are prone to quick degradation and need sophisticated delivery systems such as lipid nanoparticles (LNPs) for protection. Safety issues also exist, especially with DNA vaccines, which pose the risk of genomic integration, which can result in unwanted mutations or autoimmune responses. The poor effectiveness of mRNA and DNA vaccines against solid tumors, despite their great success against infectious illnesses like COVID-19, highlights the need for more effective strategies to penetrate the immunosuppressive tumor microenvironment, which inhibits immune activation. International distribution is also logistically challenged by complex production and storage requirements, especially the preservation of RNA vaccines at extremely low temperatures. Maximizing the effectiveness of nucleic acid vaccines as cancer immunotherapies requires overcoming these obstacles.

### Combination therapy with cancer vaccines

These cancer vaccine therapies involve using the vaccine in combination with other medications to improve the vaccine’s overall therapeutic effects. Cancer vaccines stimulate the immune system to identify and combat cancer cells, which utilize tumor-associated antigens. These therapies aim to leverage the strength of different treatments to improve outcomes, such as increasing regression of tumors, increasing survival rate, and decreasing resistance to therapy [69]. Combining gemcitabine treatment with a CTLA-4 inhibition may elicit a robust CD4 + and CD8 + T cell-mediated antitumor immune response [70]. Radiotherapy combined with CTLA-4 resulted in enhanced survival compared to radiotherapy alone. VEGF antibodies, when used alongside a tumor vaccine that promotes granulocyte–macrophage colony-stimulating factor secretion, have been shown to reduce the quantity of CD4 + CTLs and enhance vaccine efficiency [71]. PD-L1 inhibitors have demonstrated the ability to enhance the anticancer efficacy of PARP inhibitors by reinstating antitumor immunity [72]. These combination approaches are currently being investigated in cancer therapy to enhance their efficacy and address various disease-related factors. This approach has been shown to have enhanced therapeutic effects in a xenograft model, indicating that it can potentially target both tumor cells and the CSC population. One study examined carbon nanotubes conjugated with CD44 antibodies to deliver paclitaxel and salinomycin to breast cancer cells and cancer stem cells [73]. This approach is believed to contribute to treatment resistance. Additionally, another study investigated the utilization of combination antibody therapy, which is known to target the erB-2 gene in a variety of adenocarcinomas [74]. This method has accomplished substantial tumor regression in human gastric tumor cells in vitro and xenograft models. Another intriguing discovery was made in a study that assessed the role of clopidogrel in combination therapy for various cancers [75]. The study revealed a complex interaction between clopidogrel and various chemotherapeutic agents, which enhanced the efficacy of certain drugs, such as 5-fluorouracil, and reduced the efficacy of others, such as doxorubicin [76]. These results underscore the significance of evaluating potential interactions when developing combination therapies. Additionally, in a mouse model of breast cancer, a study was conducted to compare the efficacy of combination therapy, which comprises laser ablation and photodynamic therapy (PDT), to single-modality approaches [77]. This combination treatment exhibited superior results by effectively inducing tumor cell necrosis and obtaining improved management of tumor margins. In conclusion, these investigations demonstrate the potential of combination therapies to enhance the results of cancer treatment. These methods provide promising opportunities for future cancer research by focusing on various aspects of the disease and leveraging synergistic effects.

Combination cancer therapy improves treatment outcomes by targeting the tumor cells and evading resistance mechanisms. Immunotherapy, combined with cancer vaccines such as CTLA-4 and PD-L1 inhibitors, increases antitumor immune response, enhancing survival and tumor regression [78]. VEGF antibodies improve the efficiency of the vaccine, whereas PARP inhibitors regain effectiveness upon combination with PD-L1 blockade [79]. Resistance continues to pose a problem, especially through cancer stem cells (CSCs) and heterogeneity of tumors [80]. Treating CSCs with CD44-targeted nanoparticles or laser ablation combined with photodynamic therapy (PDT) has been shown to be effective in overcoming resistance [81]. Also, antibody therapy targeting the erB-2 gene and clopidogrel combinations of drugs reflects the complexity of treatment interactions [82]. Targeting resistance pathways is key to maximizing combination therapies and realizing personalized cancer therapy.

## Other promising immunotherapy

### Tumor-infiltrating lymphocyte (TIL) therapy

This therapy is a form of immunotherapy employed in the treatment of specific tumors. The process entails the extraction of tumor-infiltrating lymphocytes (TILs) from a patient’s tumor, followed by their proliferation in substantial quantities in the laboratory, and subsequently reinfusing them into the patient [83]. The therapy significantly increases these cells, hence enhancing the immune system’s ability to detect and eliminate cancer cells. CD8 + tumor-infiltrating cells are essential for a vigorous antitumor immune response [84]. Monoclonal antibodies that block immunological checkpoints to prevent T cell exhaustion and improve tumor destruction by cytotoxic CD8 + T cells are effective in mCRC patients with dMMR-MSI-H. Tumor-infiltrating lymphocytes (TILs) are essential for effective antitumor immunity, involving many T cell types, including cytotoxic T cells, T helper (TH) cells, and regulatory T cells (Tregs), which contribute to T cell-mediated immune responses inside the tumor microenvironment [35]. Researchers have demonstrated that blocking immune checkpoints significantly delays tumor growth and enhances T cell infiltration and functions; this study strongly supports the potential of co-inhibitory checkpoint blockade to improve the efficacy of TIL therapy in cancer patients. Additionally, another study suggests a potential link between Ras-MAPK pathway activation and immune invasion in triple-negative breast cancer (TNBC); this supports the exploration of MEK and PD-L1 targeted therapies in clinical trials. In a huPBL-NSG mouse model using human prostate cancer cells, TIL therapy demonstrated a predominance of CD8 + T cells with increased CD69 and CD56 expression, resulting in slowed tumor growth but not complete elimination, highlighting the model’s relevance for preclinical studies of tumor immune interactions [85]. In immunocompetent mouse models of renal adenocarcinoma and melanoma, TIL therapy using mesenchymal stem cells (MSCs) infected with oncolytic adenovirus dlE102 significantly reduced tumor volume by 50% and increased TILs, while decreasing their PD-1 + subsets demonstrates that PD-1-selected tumor-infiltrating lymphocytes (TILs) effectively recognize and target tumor cells in vivo, showing significant antitumor activity in mouse models of solid tumors and multiple myeloma, with enhanced efficacy through PDL-1 blockade [86]. Refer to Fig. 1 for how TIL therapy works.

### TCR/CAR-T cell therapy

The application of TCR-T and CAR-T cells in cancer treatment emphasizes the success in hematological malignancies and the challenges in applying these therapies to solid tumors due to the immunosuppressive tumor microenvironment and the absence of tumor-specific antigens [87]. TCR-T cells recognize intracellular and surface antigens through major histocompatibility complex (MHC) presentation, broadening their target range compared to CAR-T cells, which primarily bind extracellular antigens. However, TCR-T therapy is limited by MHC restriction, requiring patient-specific HLA matching, while CAR-T cells face difficulties in antigen escape and tumor infiltration [88]. Recent gene-editing advancements, including CRISPR/Cas9, TALENs, and zinc finger nucleases (ZFNs), have been employed to enhance T cell efficacy by knocking out endogenous TCRs, PD-1, and HLA molecules, reducing the risk of graft-versus-host disease (GVHD) and immune evasion [37]. TCR-T cells acquire antigens from the complete proteome of cells through HLAs, enabling them to identify both internal and surface antigens. TCR gene therapy is advancing the creation of optimum cellular products with minimal adverse effects and enhanced proliferative potential. Zinc finger nucleases (ZFNs) were the inaugural gene-editing technique employed to silence endogenous T cell receptors (TCRs) and generate enhanced antigen-specific T cells. ZFNs have been employed to eliminate HLA on CAR-T cells. CAR-T cells have been engineered to continuously express IL-12 and IL-15 to enhance antitumor efficacy and promote long-term persistence [89]. Additionally, armored CAR-T cells expressing costimulatory ligands (e.g., 4-1BBL, CD40L) or targeting multiple antigens through dual CAR constructs are being explored to improve tumor recognition and reduce relapse rates.[90]. Studies in preclinical models have demonstrated that CAR/TCR-T cells expressing oncolytic viruses, such as myxoma virus (MYXV), enhance tumor cell killing by inducing immunogenic cell death, addressing antigen loss and immune escape mechanisms [91]. Furthermore, clinical investigations of CRISPR/Cas9-mediated TCR knockout in anti-CD19 CAR-T cells have shown promising efficacy and safety in treating B-cell acute lymphoblastic leukemia (B-ALL), suggesting the feasibility of this approach for broader applications [92]. Despite these advances, challenges such as T cell exhaustion, trafficking to solid tumors, and off-target toxicity must be addressed to optimize the therapeutic potential of TCR-T and CAR-T cells in solid malignancies. Refer to Fig. 1 for how TCR/CAR-T cell therapy works (Table 1).

**Table 1:** Clinical studies associated with cancer

Identifier no	Target	Interventions	Outcome	Conditions
NCT01829373	T cells recognizing cancer antigens	Biological: vaccine 1650-G	Increased peripheral blood T cells recognizing cancer antigens, enhancing immune response in lung cancer	Lung Cancer
NCT04879888	Breast cancer	Biochemical: Pulsed peptide dendritic cells	Evaluates immunogenicity, safety, and side effects of the vaccine. Identifies unique tumor epitopes	Breast Cancer (Female)
NCT02285413	Melanoma tumor antigens (gp100, tyrosinase)	Biological: DC vaccines ± cisplatinum	Measures immune response to tumor antigens, clinical response, survival rates	Melanoma
NCT02261714	TG01 and GM-CSF	Biological: TG01	Assesses survival outcomes and adverse events in KRAS-mutated pancreatic cancer	Pancreatic Cancer (Resected)
NCT03481816	Metastatic castration-resistant prostate cancer (mCRPC)	Biological: adenoviral PSA, MUC1, brachyury vaccines	Evaluates safety, recommended phase 2 dose, and tumor response per RECIST 1.1	Prostate Cancer
NCT01890213	CEA protein (colon cancer)	Biological: AVX701	Assesses vaccine safety and immune response in stage III colon cancer. Results pending	Stage III Colon Cancer
NCT05062525	SARS-CoV-2 epitopes	COVID-19 Vaccine	Measures SARS-CoV-2 antibody levels and immune-related adverse events in cancer patients on immunotherapy	COVID-19, Cancer
NCT01147965	CEA protein	Biological: AD5 CEA Vaccine	Evaluates safety, immune response, and maximum tolerated dose in CEA-positive metastatic cancers	Colon, Lung, Breast Cancer
NCT01570036	HER2 + breast cancer	Drug: Herceptin, NeuVax vaccine, GM-CSF	Compares adverse events between NeuVax and GM-CSF groups	Breast Cancer
NCT03384316	MUC1, brachyury, CEA, tumor antigens	Biological: ETBX-051, ETBX-061, ETBX-011	Evaluates safety, adverse events, and dose-limiting toxicities	Colon, Prostate, Lung, Breast Cancer
NCT02692976	DC vaccines for mCRPC	Biological: mDC, pDC vaccines	Assesses safety, quality of life impact, immune response. Results pending	Prostate Cancer, Immunotherapy
NCT03014076	HER2/neu breast cancer	Drug: Trastuzumab + GM-CSF vaccine + GP2 peptide	Evaluates safety, immunologic responses, and maximum tolerated dose. Results pending	Breast Cancer
NCT03164772	NSCLC	Drug: Durvalumab, Tremelimumab, BI 1361849	Assesses safety and efficacy of vaccine plus checkpoint inhibitors	Metastatic NSCLC
NCT02380443	Metastatic colorectal cancer	Biological: AlloStim, Cryoablation	Evaluates safety, tumor response, and quality of life. Results pending	Metastatic Colorectal Cancer
NCT02115958	Cancer stem cells (CSC)	Biological: CSC vaccine	Assesses safety and immune response. Results pending	Lung Cancer
NCT02178670	CSC-primed antibodies and T cells	Biological: CSC-DC	Evaluates safety and immune response. Results pending	Ovarian Cancer
NCT02176746	CSC-primed antibodies and T cells	Biological: CSC vaccine	Evaluates feasibility of CSC-loaded DC vaccines for colorectal cancer. Results pending	Colorectal Cancer
NCT00616291	Metastatic prostate cancer	Biological: HLA class I/II peptide vaccine (NY-ESO-1/LAGE-1)	Evaluates safety, immune response, and efficacy of class II epitope vaccines. Results pending	Prostate Cancer
NCT01863108	Plasmacytoid dendritic cells (PDC)	Biological: GeniusVac-Mel4	Evaluates safety, immune response, and clinical efficacy in melanoma. Results pending	Melanoma
NCT02128126	Cervical carcinoma	Drug: ISA101/ISA101b	Assesses safety, immune response, and antitumor efficacy. Results pending	Cervical Cancer
NCT01433172	Lung cancer	Biological: GM.CD40L.CCL21 Vaccines	Evaluates safety, efficacy, and progression per RECIST criteria	Lung Cancer, Adenocarcinoma
NCT01639885	Platinum-resistant ovarian cancer	Drug: Interferon Alfa-2b, Biological: p53 SLP	Feasibility, immunogenicity, and clinical outcome assessed via CA125 levels, tumor size, and survival. Results pending	Recurrent Ovarian Cancer
NCT01678352	T cell antitumor responses in WHO grade II gliomas	Biological: Imiquimod; Tumor Lysate Vaccine	Dose-limiting toxicity, T cell response, serological responses via Western blot and flow cytometry. Results pending	WHO Grade II High-Risk Glioma, Recurrent/Post-Chemotherapy Glioma
NCT01376505	Tumor cells	Scientific: HER2 vaccines, Biological: OBD’s HER2 vaccine	Immune response, safety, toxicity (CTCAE v4.0), HER2 immune response kinetics. Results pending	Breast Cancer, Malignant Colon GIST Tumor, Ovarian Cancer
NCT01922921	Stage IV HER2 + breast cancer	Biologicals: HER2/neu Protein, Polysaccharide-K, Trastuzumab, Etanercept, Placebo	Toxicity and adverse event monitoring. All patients experienced some adverse events	Stage IV HER2 + Breast Cancer
NCT00157209	Stage IIIB/IV NSCLC	Biological: Tecemotide (L-BLP25), Drug: Cyclophosphamide, Best Supportive Care (BSC)	Immune response, quality of life, antigen CA27-29 levels. Adverse events occurred in all patients	Non-Small Cell Lung Cancer
NCT00601796	Stage IV lung adenocarcinoma	Biochemical: Immunization, Drug: Cyclophosphamide, ATRA	Survival, progression-free survival, immune system response. 100% experienced adverse events	Lung Cancer
NCT00643097	Glioblastoma multiforme	Biological: PEP-3 vaccine, Sargramostim, Temozolomide	Progression-free survival, immune response, toxicity. 94–100% had adverse events	Brain Malignant Neoplasms
NCT01417000	Metastatic pancreatic cancer	Biological: GVAX Pancreas, CRS-207, Drug: Cyclophosphamide	Safety, immune response, treatment effects. 100% experienced adverse events	Metastatic Pancreatic Cancer
NCT00005947	Metastatic hormone-refractory prostate cancer	Biological: Sipuleucel-T, Placebo	Monitored for disease progression. 97–100% had adverse events	Prostate Cancer
NCT03391232	Cancer cells	Biological: PolyPEPI1018 CRC Vaccine	Personal epitope response, immune reactions. 40–100% had adverse events	Colorectal Cancer
NCT00103142	Tumor cells	Biologicals: Falimarev, Inalimarev, Sargramostim, Dendritic Cells	Immune response (ELISpot analysis). 97–100% experienced adverse events	Colorectal Cancer, Metastatic Cancer
NCT00583024	Prostate cancer cells	Biological: ADENOVIRUS/PSA VACCINE	Vaccine response, PSA levels. 56–100% had adverse events	Hormone-Refractory Prostate Cancer
NCT00583752	Prostate cancer cells	Biological: Adenovirus/PSA Vaccine	Immune response, survival rates. 60–72% had adverse events	Recurrent Prostate Cancer
NCT00194714	HER2-specific immunity	Biological: HER2/neu Peptide Vaccine	HER2 immune response, biomarker analysis. 100% experienced adverse events	Stage IV Ovarian and Breast Cancer (HER2/Neu +)
NCT02193347	Grade II primary brain tumor	Bio: PEPIDH1M Vaccine, Tetanus-Diphtheria, Drug: Temozolomide	Toxicity assessment, immune response (IFNγ ELISpot). 100% had adverse events	Brain Tumor
NCT02063724	HER2 high/intermediate breast cancer	Biological: Pulsed Dendritic Cell Vaccine for HER2	Immune response, mammogram changes. 66.67% had adverse events	Breast Cancer
NCT02648282	Cancer cells	Drugs: Cyclophosphamide, GVAX Pancreas Vaccine, Pembrolizumab, SBRT radiation	Metastasis-free survival, immune toxicity monitoring. 93.10% had adverse events	Pancreatic Cancer
NCT01245673	Myeloma cells	Biologicals: Prevnar, Activated T cells, Lenalidomide, MAGE-A3/GM-GSF	T cell response, paraprotein levels. 100% had adverse events	Myeloma
NCT01867333	Cancer cells	Biologicals: PROSTVAC, TRICOM, Enzalutamide	Lesion progression, PSA levels. 100% had adverse events	Prostate Cancer
NCT04516330	Breast tissues	Genetic: miRNA	MicroRNA analysis to guide breast cancer treatment. Results pending	Multiplex Breast Cancer
NCT03591367	Non-muscle-invasive bladder cancer	Diagnostic Test: RT for telomerase, MicroRNAs-155	Serum vitamin D correlation with bladder cancer. Results pending	Bladder Cancer
NCT01964508	Malignant and benign lesions	Other: Fine needle aspiration	MicroRNA diagnostic potential for thyroid cancer. Results pending	Thyroid Cancer
NCT03824613	Urinary miRNA in endometrial cancer	Diagnostic Test: Urinary miRNA	MicroRNA as a diagnostic marker for endometrial cancer. Results pending	Endometrial Cancer
NCT01612871	Breast cancer	Drug: Tamoxifen, Letrozole, Anastrozole, Exemestane	Evaluates plasma miRNAs before and after treatment to correlate with therapy response	Breast Cancer
NCT00806650	Renal cell carcinoma (RCC)	Genetic and biomarker analysis	Assesses IMP3 autoantibody test sensitivity using IHC as a standard	Renal Cancer
NCT02471469	Metastatic prostate cancer	Drug: Enzalutamide	Investigates biomarker response to therapy for personalized treatment	Prostate Cancer
NCT03293433	Lung nodules (benign or early-stage cancer)	Blood sampling	Evaluates 34 miRNA extraction methods for consistency and diagnostic value	Lung Cancer
NCT05815407	Non-small cell lung cancer	No intervention	Analyzes diagnostic and prognostic value of miRNAs 106b-5p, 601, and 760	Lung Cancer
NCT01572467	Testicular or ovarian sex cord stromal tumors	Genetic and biomarker analysis	Correlates DICER1 mutations with miRNA pathways and outcomes	Ovarian and Testicular Cancer
NCT02412579	Hepatocellular carcinoma (HCC)	Standard of Care	Identifies liver cancer-specific genetic markers via next-gen sequencing	Hepatocellular Carcinoma
NCT01528956	Pediatric adrenocortical tumors	Genetic and RNA analysis	Identifies genetic alterations in adrenocortical tumor samples	Adrenocortical Carcinoma
NCT03693703	Prostate cancer (PCa)	Bi-parametric and Multiparametric MRI	Compares MRI methods and develops a decision support system using miRNA assessment	Prostate Cancer
NCT01229124	Acute leukemia	Genetic and biomarker analysis	Identifies microRNA sequences linked to AML in infants	Leukemia
NCT01057199	AML with CEBPA mutations	Genetic and biomarker analysis	Studies miRNA-34a and miRNA-194 roles in granulopoiesis and myeloid cell proliferation	Leukemia
NCT01482260	Cutaneous malignant melanoma	Biopsy	Uses microarray and RT-PCR to profile miRNA expression	Melanoma
NCT01298414	Pediatric AML and MSC niche	Genetic and biomarker analysis	Examines miRNA expression changes in AML cells within the MSC niche	Leukemia
NCT04913545	Malignant transformation in oral lesions	Salivary miRNA test	Evaluates salivary miRNAs (412,512) for early cancer detection	Oral Premalignant Lesions
NCT01916239	Colorectal cancer	Pomegranate extract	Analyzes miRNA expression, gene profiles, and metabolic effects in colon tissues	Colorectal Cancer
NCT01511575	AML and transient myeloproliferative disorders in Down syndrome	Genetic and biomarker analysis	Compares gene expression in DS and non-DS AML patients	Leukemia
NCT05449847	Hepatosteatosis and liver fibrosis	Blood sampling	Assesses relationship between MicroRNA and HCV	Hepatitis C

### Immune checkpoint inhibitors

A specific form of medication inhibits the production of checkpoint proteins by immune system cells, including T cells and certain cancer cells. These checkpoints, such as PD-1/PD-L1 and CTLA-4/B7-1/B7-2, regulate immune responses but can also prevent T cells from destroying tumor cells[93]. Immune checkpoint inhibitors (ICIs) block these interactions, allowing T cells to remain active against cancer PD-L1 on tumor cells binds to PD-1 on T cells, suppressing immune responses. ICIs targeting PD-1 (anti-PD-1) or PD-L1 (anti-PD-L1) restore T cell function by preventing this binding [94]. Similarly, CTLA-4 on T cells binds to B7-1/B7-2 on antigen-presenting cells (APCs), maintaining immune suppression. Anti-CTLA-4 antibodies disrupt this interaction, enabling T cell activation [95]. ICIs have significantly advanced cancer immunotherapy by removing inhibitory immune checkpoints and promoting robust antitumor responses [37]. The US Food and Drug Administration (FDA) has approved ICIs targeting three checkpoint molecules: CTLA-4, PD-1, and PD-L1. Ipilimumab, the first anti-CTLA-4 antibody, was approved for metastatic melanoma [96]. Anti-PD-1 antibodies, which block PD-1 from interacting with PD-L1 and PD-L2, further enhance T cell responses. The FDA has also approved three anti-PD-L1 antibodies—atezolizumab, durvalumab, and avelumab—for cancers such as urothelial carcinoma, non-small cell lung cancer (NSCLC), and Merkel cell carcinoma [97]. Additionally, a study demonstrated that the combination of anti-PD-1 therapy with PSMA-targeted radionuclide therapy significantly improved disease control (TTP: 47.5 days, survival: 51.5 days) in a C57BL/6 mouse model of prostate cancer, compared to monotherapies [98].

## MicroRNA’s in cancer

**Fig. 2:**
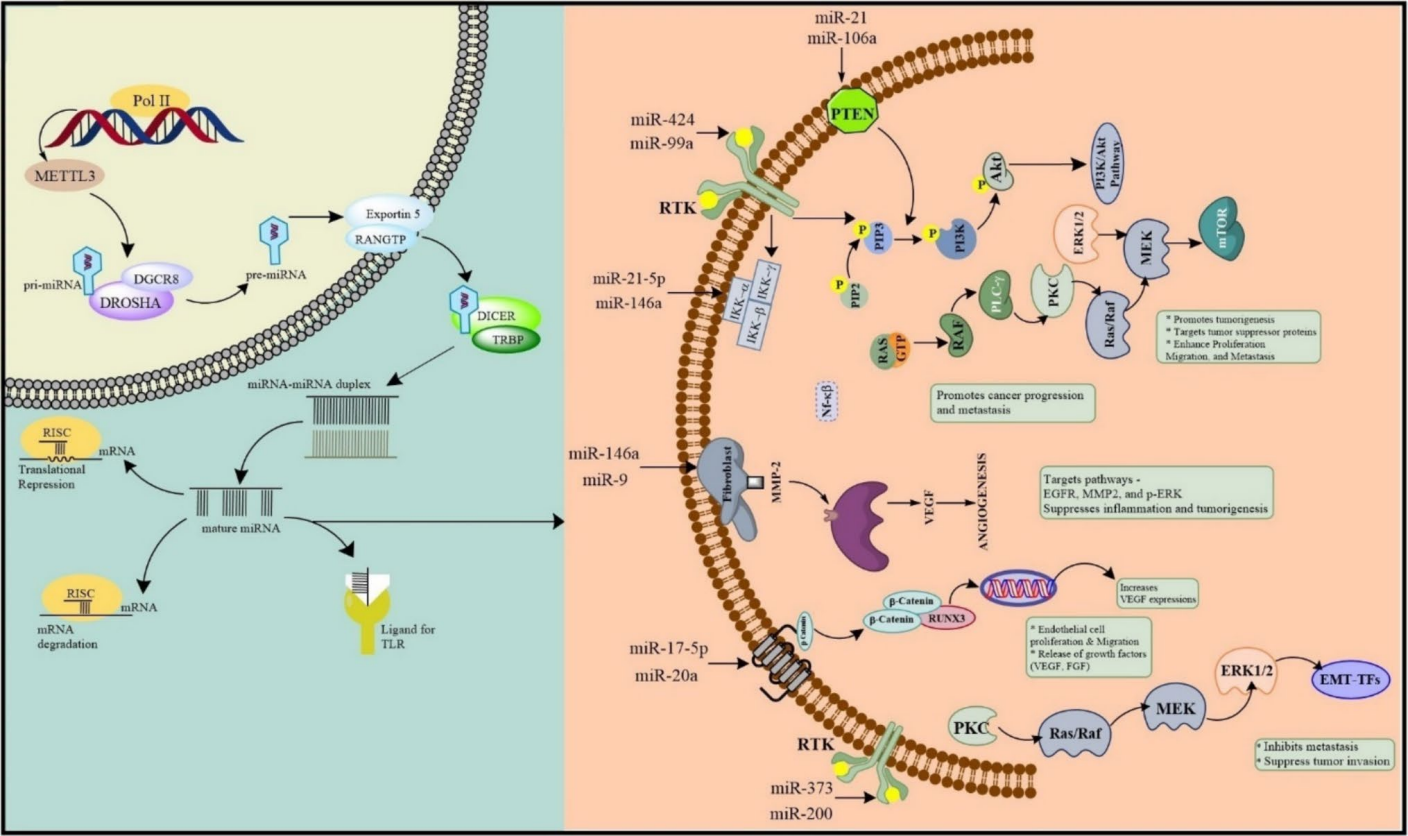
Schematic illustrates the critical roles of various miRNAs in cancer progression by targeting key signaling pathways and biomarkers. miR-99a and miR-424 suppress the PI3K/AKT/mTOR pathway, which regulates cell proliferation and survival, while miR-21-5p modulates the NF-κB pathway, promoting inflammation and tumor progression. miR-106a influences tumorigenesis by regulating PTEN, a crucial tumor suppressor

A class of small non-coding RNAs known as miRNAs is involved in post-transcriptional gene silencing [99]. MicroRNAs are a subclass of non-coding RNAs that control the expression of genes by either translatory inhibition or mRNA degradation. About half of miRNAs are autonomously transcribed and encoded on non-protein-coding regions. The introns of transcripts that code for proteins contain the remaining miRNAs. Tumor suppressor miRNAs and oncogenic miRNAs, also known as oncomiRs, are two types of miRNAs that are important in cancer development [100]. A multitude of human miRNA genes are positioned in genomic regions that are deleted, amplified, or translocated in cancer, or situated at fragile locations. The transcription of pri-miRNA represents the initial phase of miRNA synthesis and is dysregulated in several human malignancies [101]. These genetic alterations influence miRNA and pri-miRNA transcription, resulting in the abnormal production of downstream target mRNAs that may promote cancer formation and dissemination. The initial example originated from the induced expression of the miR-17 ~ 92 cluster, referred to as oncomiR-1, which collaborated with MYC to accelerate tumor growth in a B-cell lymphoma murine model [102]. MiRNA’s canonical biogenesis is a multi-step process involving cytoplasmic and nuclear phases. A single nuclear miRNA gene, transcribed by RNA polymerase II (Pol II), produces a hairpin intermediate termed “pri-miRNA,” which is subsequently identified by a microprocessor primarily consisting of two molecules of DiGeorge syndrome critical region gene 8 (DGCR8), a cofactor of Drosha, an RNase III enzyme featuring two RNase III domains, and one molecule of Drosha. Drosha subsequently cleaves the stem of the pri-miRNA hairpin into two strands, resulting in a stem-loop structure referred to as “pre-miRNA.” Exosomes can encapsulate miRNAs, subsequently participating in intercellular communication [99]. The initial evidence of miRNAs’ involvement in human cancer emerged in 2002, when B-cell chronic lymphocytic leukemia (B-CLL) was associated with the deregulation of miR-15a and miR-16-1. The greatest biomarker for doxorubicin-induced cardiotoxicity was found to be miR-1, which exhibited significantly higher expression than other miRNAs and improved the assessment of cardiac injuries in patients with cardiotoxicity as opposed to cardiac troponin I (cTnI) [103]. The extensive deregulation of miRNAs in human cancers shown in recent decades underscores the critical role of miRNAs in the development, metastasis, and onset of tumors. A global reduction in miRNA expression in tumor cells compared to normal cells has been documented, as demonstrated by the profiling of 217 mammalian miRNAs from both normal and human cancer specimens. Apart from the overall reduction in miRNA expression, it has been demonstrated that 540 solid tumor samples had differentially expressed miRNAs. This indicates that specific alterations in each miRNA expression were evident in tumors, as miRNA expression becomes unregulated during cancer progression, leading to a different expression pattern. In the initial phases of diffuse large B-cell lymphoma (DLBCL), miR-21 expression is elevated relative to subsequent stages. Hypoxia, characterized by diminished oxygen levels in the tumor microenvironment (TME), might influence the maturation and functionality of mature microRNAs (miRNAs) due to the regulation of miRNA expression and function by cellular stress. The hypoxic environment stimulates epidermal growth factor receptor (EGFR) signaling to promote growth and oncogenesis. MicroRNAs are essential for the regulation of cellular activities, including cell proliferation, metabolism, and protein synthesis, in normal physiological conditions. Their dysregulation causes aberrant cell growth and biosynthesis, promoting tumors’ development, spread, and metastasis [104]. Any miRNA deregulation will contribute to the growth of tumors. MiRNA expression profiles have the potential to be a useful noninvasive diagnostic tool. Only cancerous tissues and cell lines showed upregulation of miRNA-93, miRNA-196a, miRNA-196b, miRNA-203, miRNA-205, miRNA-210, miRNA-221, miRNA-222, and miRNA-224. MiRNAs can potentially be ground-breaking gene therapies, but their effectiveness largely depends on a reliable delivery system [100]. Exosomes have been shown to impact several aspects of cancer, such as EMT, multidrug resistance, metastasis, and progression. It has been reported that miRNAs impact immune responses against tumors and tumor immunogenicity. For instance, a study found that miR-124-3p is the target of LINC00240 and may stimulate the development of cervical cancer through the actions of MHC class I-related proteins A (MICA) and STAT3, which in turn mediates the tolerance of natural killer T (NKT) cells [99]. According to reports, miR-449a plays a role in carcinoma development and could be a useful prognostic marker. We believe that miR-449a can potentially serve as a therapeutic agent for treating certain types of cancer based on the putative pathogenetic relationships between cancer and miR-449a [105]. miRNAs may be useful instruments for prognostication and diagnosis to enhance cancer prognosis. One of the most active cell signaling pathways in cancer is PIK3/AKT/MTOR [103] (Fig. 2). The following evidence proves that miRNA profiling holds diagnostic potential while standardization of clinical application lacks. Moreover, the accuracy of miRNAs as noninvasive biomarkers is encouraging but needs further validation in a heterogeneous population, and there is a need for additional research to interpret efficacy and safety of miRNA therapy in human trials. Nonetheless, recent findings reveal that extracellular vesicle-delivered miRNA transfer can remold tumor microenvironment, and it has therapeutic potential. It has also been shown that miRNA expression is regulated by tumor metabolism, which may be targeted for the treatment of cancer. CRISPR-based gene editing has opened up avenues to precisely manipulate miRNAs and has the potential for personalized medicine.

### Oncomirs and oncosuppressive microRNAs

Oncomirs are microRNAs associated with cancer, categorized based on their roles in various tumorigenic processes [106]. A key group of oncomirs, including miR-21, miR-181a, miR-632, and miR-221/222, are implicated in metastasis, while another category, consisting of miR-155 and miR-375, drives the proliferation of neoplastic cells [107]. MicroRNAs such as miR-182, miR-10b, miR-373, and miR-520c are critical for tumor cell invasion into organs, and miR-9 and miR-27a are involved in angiogenesis, aiding tissue vascularization [108]. Additionally, miR-22, miR-181a, and miR-221/222 participate in tumor progression. In contrast, the let-7 family of microRNAs acts as a significant tumor suppressor, with its overexpression in early-stage bladder cancer inhibiting the activity of oncogenes like H-RAS and HMGA2 [109]. Another well-studied suppressor microRNA, miR-145, is typically expressed at lower levels in cancer cells compared to normal tissues [110]. The miR-200 family, comprising miR-200a, miR-200b, miR-200c, miR-429, and miR-141, plays a vital role in the invasion of solid tumors, highlighting the diverse yet critical functions of microRNAs in cancer biology [111] (Fig. 2).

Although enormous progress has been made in miRNA study, some gaps exist. The intricate relationship between oncomirs and tumor suppressor miRNAs between distinct cancer subtypes needs further probing to establish their specific roles during tumorigenesis. Specifically, miR-375 shows conflicting behavior in different cancers, and its function needs to be subjected to a more in-depth exploration. Lastly, the long-term consequences of miRNA-based therapeutic interventions in cancer therapy are poorly understood, and it is essential to conduct extensive preclinical research. Conversely, recent findings indicate that miRNA expression is epigenetically controlled, allowing for new epigenetic therapies. In addition, combination therapies that include miRNA modulators and immune checkpoint inhibitors show synergistic effects, increasing the potential for miRNA therapeutics. Finally, identifying cooperative miRNA clusters provides new avenues for combination therapy as targeted agents, providing a more nuanced method for cancer treatment. These observations highlight the need for ongoing studies to fill existing gaps and refine miRNA-based oncology approaches.

### The role of miR-99a

miR-99a has been identified as a tumor suppressor gene in multiple human malignancies [112]. The tumor suppressor gene miR-99a is often deleted or expressed at diminished levels in numerous human malignancies. For instance, miR-99a was observed to be downregulated in esophageal squamous cell carcinoma tissues, and diminished miR-99a expression was associated with poorer overall patient survival. Transient gene transfection of miR-99a overexpression suppressed esophageal cancer cell proliferation and triggered apoptosis [112]. miR-99a can selectively suppress IGF1R expression, hence inhibiting the proliferation and migration of cervical cancer cells (CCCs) [113].

miR-99a has emerged as a multifaceted tumor suppressor with significant roles in various cancers, including non-small cell lung cancer (NSCLC). Studies highlight its therapeutic potential, demonstrating that miR-99a overexpression enhances radiosensitivity in NSCLC, improving radiation efficacy and suggesting its role in better treatment outcomes [114]. Additionally, miR-99a acts as a tumor suppressor in NSCLC by inhibiting cell migration and invasion through AKT1 downregulation [115]. However, its role appears context-dependent, as research in breast cancer shows miR-99a and miR-99b functioning as downstream targets of TGF-β, promoting epithelial-to-mesenchymal transition (EMT) by increasing cell migration and fibronectin expression while reducing E-cadherin and ZO-1 [116]. This duality underscores its potential contribution to tumor progression in certain contexts. In endometrial cancer (EC), miR-99a is notably downregulated in tumor tissues, correlating with poor differentiation and aggressive behavior [117]. Overexpression studies reveal its ability to inhibit cell proliferation, induce apoptosis, suppress invasion, and curb tumor growth in vivo, primarily through dual suppression of the PI3K/AKT/mTOR pathway [118]. These findings establish miR-99a as a promising biomarker and therapeutic target across different cancer types (Fig. 2).

In spite of exhaustive studies on miR-99a, there are some gaps that exist in completely unraveling its cancer role. Its dual role as a tumor promoter and suppressor in various forms of cancer warrants further studies to understand its context-dependent functions. Furthermore, the exact mechanisms by which miR-99a controls the PI3K/AKT/mTOR pathway in various cancers are yet unknown, which makes further molecular investigations necessary. From a clinical point of view, although miR-99a holds promise as a biomarker, its validation has just begun and highlights the need for more cohort studies to confirm its diagnostic and prognostic credibility. Emerging information, though, points toward its new potential as a therapy. Experiments indicate that miR-99a boosts radiosensitization, bringing new hope to combination therapies, especially in lung cancer. Additionally, recent research suggests that miR-99a is involved in regulating cancer cell metabolism, offering a new area for targeted interventions. Another important finding is that tumor hypoxia controls the expression of miR-99a, linking it to the tumor microenvironment and further increasing its significance in cancer development and therapeutic approaches. These new findings underscore the necessity of further investigation to realize miR-99a’s full potential in cancer therapy.

### The role of miR-21-5p

microRNA appears frequently upregulated in various cancers, promoting cell viability, proliferation, and migration while inhibiting apoptosis in many cases. Several studies have shed light on the complex role of miR-21-5p in cancer [119]. MiR-21-5p exhibited elevated expression across multiple cancer types. MiR-21-5p enhanced cell survival, proliferation, and migration while inhibiting apoptosis by targeting BTG2. The MiR-21-5p inhibitor diminished the size and volume of xenograft tumors, as well as the expressions of Ki67 and miR-21-5p in the xenograft tumor tissues [120]. miR-21-5p was both overexpressed and repressed in cancer stem cells (CSCs) [121]. Inhibition of miR-21-5p in melanoma cell lines diminishes cell proliferation and enhances apoptosis via elevating the expression of PDCD4, PTEN, and BTG2. miR-21 can additionally associate with toll-like receptor 8, resulting in toll-like receptor-mediated activation of NF-kB [122].

Furthermore, some studies identify exosomal miR-21-5p as a key player in promoting cancer. The authors demonstrate that exosomal miR-21-5p from cancer cells induces mesothelial-to-mesenchymal transition (MMT) in peritoneal mesothelial cells, facilitating metastasis [123]. This effect was mediated through targeting SMAD7 in the TGF-β/Smad pathway, validated by in vitro and in vivo models. These findings suggest that targeting exosomal miR-21-5p could be a promising therapeutic strategy for gastric cancer. Another study investigated the role of exosomal miR-21-5p in colorectal cancer (CRC) [124]. Exosomal miR-21-5p from CRC cells promoted angiogenesis and vascular permeability by targeting KRIT1 and activating the β-catenin pathway in endothelial cells [125]. Notably, higher exosomal miR-21-5p levels were observed in CRC patients compared to healthy controls, highlighting its potential as a diagnostic or therapeutic target [126]. Furthermore, the review discusses Andrographolide (Andro), a natural compound with antitumor properties. In a transgenic breast cancer model, Andro inhibited tumor growth and metastasis by suppressing proliferation, migration, and invasion of cancer cells [127]. Mechanistically, Andro suppresses NF-κB activation, leading to downregulation of miR-21-5p expression and promotion of PDCD4, a tumor suppressor [127]. These findings suggest that Andro may be a promising therapeutic agent for luminal-like breast cancer by targeting the miR-21-5p pathway (Fig. 2). Although miR-21-5p has been extensively studied, there are still some knowledge gaps regarding its function in cancer. Its regulation of tumor immunity is not fully explored, so concerns exist regarding its contribution to immune evasion and response. Furthermore, though miR-21-5p has the potential to serve as a liquid biopsy biomarker, it still requires validation before it can be considered clinically useful. Another key field to be studied is the exact mechanisms by which miR-21-5p is involved in drug resistance, which are still not well understood. New findings have clarified its function in enhancing metastasis by exosomal communication, with implications for cancer development. Also, new data indicate that natural products such as Andrographolide may suppress miR-21-5p, providing new potential therapeutic approaches. Moreover, its NF-κB regulation connects miR-21-5p to inflammatory tumor signaling, again highlighting its cancer pathophysiology role. Closing these research gaps may open new avenues for targeted therapies and biomarker discovery in cancer treatment.

### The role of miR-4728

miR-4728 was recently discovered within an intron of the ErbB2 gene. mir-4728 is a tumor-suppressive miRNA that regulates MAPK signaling through the targeting of MST4, highlighting its potential as a prognostic biomarker and therapeutic target in oncology [128]. The pre-miR-4728 (mir-4728) is encoded within an intron of the HER2 gene. Patients with HER2 positivity exhibiting elevated miR-4728 expression levels demonstrated improved responsiveness to targeted treatments [129]. miR-4728 was the predominant independent risk factor for tumor recurrence in patients with advanced T stage [129]. miR-4728 exhibits dual roles in HER2 + breast cancer, acting as both an oncogenic factor and a tumor suppressor depending on the context. Studies have shown that miR-4728 can contribute to resistance against HER2 inhibitors like lapatinib by downregulating NOXA expression through reduced ESR1 transcription, with pharmacological inhibition of MCL-1 emerging as a potential strategy to overcome this resistance [130]. Conversely, the miR-4728-5p variant has been identified as an oncogenic factor that promotes proliferation and migration in breast cancer cells by targeting EBP1, creating a feedback loop that amplifies HER2 signaling [131]. On the other hand, miR-4728 also demonstrates tumor-suppressive properties by inhibiting the MAPK pathway via MST4 targeting, with its downregulation in breast tumors correlating with poor patient prognosis [129]. This dual functionality highlights its potential as both a therapeutic target and a prognostic marker, emphasizing the need for context-specific strategies when designing miR-4728-focused therapies. (Figure 2) Notwithstanding increasing evidence of the role of miR-4728 in HER2 + cancers, much research remains to be done. Its dual oncogenic and tumor-suppressive activities must be explored further to understand its exact role in tumor development. Moreover, the long-term consequences of targeting miR-4728 in HER2 + breast cancer are unknown, and more studies need to be conducted to assess possible therapeutic risks and benefits. A further important void is in identifying if miR-4728 would be a universal prognostic marker for all cancer types. Nevertheless, some recent findings indicate its potential in drug resistance reversal, as miR-4728 has been reported to regulate resistance to HER2 inhibitors. Additionally, its ability to control the MAPK pathway suggests a larger role beyond breast cancer, possibly extending to other cancers. Significantly, the finding that miR-4728-5p is involved in the migration of tumor cells further enhances its potential as a therapeutic target, highlighting the necessity for further investigation of its functions in the development of cancer and in treatment protocols.

### miR-17-5p, miR-20a, and miR-106a

miR-17-5p may serve as a novel tumor marker for monitoring cancer development and progression. miR-17-5p exhibits a predictive influence on several tumor types [132]. miR-17-5p is intricately linked to malignancies via complex regulatory pathways [133]. miR-17-5p is highly overexpressed in all tumor types. Pterostilbene downregulated the expression of miR-17-5p and miR-106a-5p in tumors and systemic circulation, restoring PTEN mRNA and protein levels, which resulted in decreased tumor growth in vivo [134]. miR-20a’s function in regulating breast cancer angiogenesis suggests its potential as an angiogenic biomarker [135]. Elevated expression levels of miR-20a have been detected in colorectal cancer (CRC) tissues, serum, and plasma. miR-20a was shown to be present in feces and demonstrates excellent sensitivity for colorectal cancer (CRC). Consequently, miR-20a may serve as a biomarker for colorectal cancer (CRC) and an indicator to avert invasive procedures in affected patients. miR-20a demonstrates potential for clinical application as a novel diagnostic biomarker and therapeutic target for colorectal cancer patients [136]. miRNA modulates the expression of essential cell cycle and apoptosis components, indicating miR-106a as a promising diagnostic and prognostic biomarker with therapeutic implications. Levels of miR-106a are highly correlated with tumor stage, size, differentiation, lymphatic and distant metastases, and invasion [137]. MicroRNAs (miRNAs) play a critical role in cancer progression, with several emerging as potential diagnostic markers or therapeutic targets. For instance, miR-17-5p has been shown to directly target RUNX3 in gastric cancer (GC), leading to enhanced cell proliferation and invasion. Studies using mouse models revealed that miR-17-5p overexpression significantly increased tumor growth, while its inhibition reduced tumor volume, making it a promising therapeutic target for GC [133]. Similarly, in nasopharyngeal carcinoma (NPC), miR-17-5p promotes tumor progression by downregulating p21, a key cell cycle inhibitor. This downregulation drives increased cell proliferation and tumor growth, as demonstrated in both in vitro studies and in vivo xenograft models, further highlighting miR-17-5p’s potential as a diagnostic or therapeutic tool in NPC [138]. Beyond miR-17-5p, miR-20a displays a context-dependent role in breast cancer, particularly in angiogenesis. In vitro experiments showed that miR-20a enhances blood vessel formation in MCF7 cells but inhibits it in MDA-MB-231 cells [135]. Clinically, higher miR-20a expression has been associated with larger blood vessels and increased VEGFA expression in invasive breast carcinomas. These findings underscore the complexity of miR-20a’s role in angiogenesis, necessitating further research to define the conditions under which it promotes or inhibits tumor vascularization [135]. In non-small cell lung cancer (NSCLC), miR-106a overexpression has been linked to aggressive cancer phenotypes by suppressing PTEN, a tumor suppressor. This suppression drives increased cell proliferation, migration, and invasion, as observed in NSCLC cell lines and tissues [139]. Given its role in facilitating tumor progression, miR-106a represents a compelling target for therapeutic interventions in NSCLC. Collectively, these studies emphasize the critical role of miRNAs like miR-17-5p, miR-20a, and miR-106a in cancer progression and highlight their potential as targets for innovative treatment strategies (Fig. 2).

Anyhow the vast research on miR-17-5p, miR-20a, and miR-106a in cancer, there are some gaps in knowledge regarding their exact roles in tumorigenesis. The molecular pathways by which miR-17-5p promotes tumorigenesis in various cancers are poorly defined, restricting the scope for specific therapeutic targeting. Likewise, functional variation in the angiogenic roles of miR-20a in different tumor microenvironments needs to be explored, given that its action on tumor vascularization seems to be dependent on context. Further, the role of miR-106a in immune modulation in cancer is also less understood, which creates a difficulty in fully interpreting its role in tumor growth. Recent evidence indicates that miR-17-5p is a promoter of tumor growth through targeting major suppressors like RUNX3, which offers new therapeutic possibilities. Further, the differential influence of miR-20a on angiogenesis reflects the multifaceted nature of its action in tumor biology. In contrast, miR-106a has been recognized as a tumor promoter through PTEN downregulation and is a potential target for targeted therapy. Filling these gaps in research will be important to the development of effective miRNA-based cancer therapies.

### miR-155

miR-155 is a highly conserved and versatile microRNA, primarily distinguished by its overexpression in several illnesses, particularly malignant tumors. The modified expression of miR-155 is linked to numerous physiological and pathological processes, such as hematopoietic lineage differentiation, immunological response, inflammation, and cancer [140]. miR-155, a non-coding microRNA, is revealed to have a strong correlation with various nutritional processes [141]. MicroRNA-155 (miR-155) is a versatile regulator of cell proliferation, the cell cycle, development, immunity, and inflammation, playing crucial and occasionally contradictory roles in various malignancies, including esophageal cancer [142]. One study has investigated its function in metastasis using a metastasis cell line model in immunodeficient mice. Interestingly, miR-155 overexpression in highly metastatic CL16 cancer cells significantly decreased lung tumor burden. This effect appears to be driven by reduced cell invasiveness without affecting proliferation or apoptosis in established tumors [143]. These findings suggest a potential therapeutic role for miR-155 in targeting metastasis without compromising overall tumor growth. Another study explored miR-155 in the context of pancreatic cancer, utilizing the p48-Cre/LSL-KrasG12D mouse model. Here, miR-155 levels increased during malignant progression, accompanied by a downregulation of its predicted target, MLH1. Additionally, this study observed distinct changes in circulating miRNAs following gemcitabine treatment, suggesting potential links between miR-155 and drug response [144]. While the precise role of miR-155 in pancreatic cancer progression remains to be elucidated, this study highlights its potential involvement and the influence of treatment on miRNA expression profiles (Fig. 2). Although there has been extensive research, the function of miR-155 in metastasis is unclear, and more in vivo experiments are needed to determine its role in various cancers. Its role in drug resistance in pancreatic cancer is also poorly understood, calling for more targeted studies. The clinical use of miR-155 suppression remains to be established, despite the fact that it has been identified as a possible treatment strategy. Remarkably, new research suggests that overexpressing miR-155 may reduce metastasis in some cancer types, suggesting potential therapeutic benefits. Gemcitabine treatment has also been demonstrated to modify circulating miRNAs, including miR-155, which may impact how the medicine works. Further highlighting its significance in tumor growth and treatment response, miR-155 may potentially modulate the immune response in cancer due to its participation in regulating inflammatory signaling pathways.

### miR-424

MiR-424’s position in cancer may serve as a prognostic and diagnostic biomarker and a possible therapeutic candidate for cancer treatment. Mature miR-424 induces the degradation of its target transcripts or inhibits translation by binding to molecular targets. miR-424 participates in the modulation of p53, PI3K/Akt, Wnt, and various other molecular pathways, governing cellular proliferation, apoptosis, differentiation, chemoresistance, and cancer immunity [145]. miR-424 has been identified as a tumor-suppressive microRNA in certain cancer types, functioning as an oncogene in others. miRNA is upregulated in melanoma, laryngeal and esophageal squamous cell carcinomas, glioma, multiple myeloma, and thyroid carcinoma [146]. Melatonin exerts a tumor-suppressive effect by modulating the miR-424-5p/VEGFA axis. miR-424 has been demonstrated to facilitate apoptosis and induce cell cycle arrest in glioblastoma cells [147]. miR-424-5p can enhance proliferation and metastatic-related characteristics by directly interacting with SCN4B [148]. miR-424(322)/503 is a tumor suppressor in breast cancer. A lack of miR-424 causes tumors to form and resistance to chemotherapy to develop [149]. Research using a knockout mouse model and primary tumor analysis shows its promising potential as a therapeutic target. On the other side, circTBL1XR1 a circular RNA with the opposite effect in colon cancer [150]. When overexpressed, it ramps up cancerous behaviors like growth, migration, and invasion. But when it is dialed down, these effects are reversed. circTBL1XR1 seems to target and suppress Smad7, which may help explain its role in advancing colorectal cancer (Fig. 2). Despite being widely studied regarding miR-424, little is known regarding its dual functions as an oncogene and a tumor suppressor in various forms of cancer and the mechanisms that mediate its chemoresistance. Further research is therefore necessary to clarify this function. Another key lacuna is the paucity of investigation into miR-424’s role in the immune response in the tumor microenvironment, which would be of great interest in understanding its overall regulatory roles. Recent evidence has highlighted novel facets of miR-424 regulation, including melatonin-mediated regulation of the miR-424-5p/VEGFA axis, with implications for new combination therapies. Reiterating its function in the formation of cancer, miR-424(322)/503 is also known to be a tumor suppressor in breast cancer, and its loss is what causes chemoresistance. Furthermore, the control of miR-424 by circTBL1XR1 in colorectal cancer demonstrates the novel function of circular RNAs in miRNA modulation, opening the door to novel targeted treatment strategies.

### miR-146a

miR-146a-5p may serve as a noninvasive biomarker and a targeted therapy in certain malignancies. Direct targets of miR-146a are overexpressed, resulting in enhanced cell proliferation, invasion, metastasis, and survival. miR-146a-5p generated from cancer-associated fibroblasts can enhance stemness and increase chemoresistance in urothelial bladder cancer. Exosomal miR-146a-5p may serve as a biomarker for UBC recurrence and a prospective therapeutic target [151]. CAFs transfected with miR-146a-5p demonstrated a significant elevation in the levels of inflammatory cytokines interleukin-6, tumor necrosis factor-α, transforming growth factor-β, and CXCL12, which initiate the epithelial-mesenchymal transition and pro-metastatic transformation of colorectal cancer cells [152]. Crucially, the activation of cancer-associated fibroblasts (CAFs) by miR-146a-5p promoted tumorigenesis and pulmonary metastasis of colorectal cancer (CRC) in vivo through tumor xenograft models [153].

Researchers, using the KCI transgenic mouse model, discovered that a decrease in miR-146a contributes to the overexpression of the epidermal growth factor receptor (EGFR) [154]. Treating these mice with CDF—a synthetic compound—brought miR-146a levels back to normal. This reduced EGFR levels and slowed tumor growth in xenograft models. Restoring miR-146a with CDF could be a game-changer for personalized pancreatic cancer treatments. In castration-resistant prostate cancer (CRPC), miR-146a levels drop significantly compared to androgen-dependent prostate cancer (ADPC) [155]. In in vivo as well as in vitro studies show that boosting miR-146a levels slowed down cancer cell growth, migration, and tumor formation in androgen-independent prostate cancer cell lines [156]. It seems this works by suppressing EGFR and MMP2 and dialing down p-ERK signaling. All of this points to miR-146a’s role in controlling CRPC progression. Research with genetically engineered mice has revealed that removing miR-146a leads to serious immune system issues, such as increased inflammation, overactive myeloid cell production, and tumor development [157]. In short, miR-146a is a natural regulator, keeping inflammation, cell proliferation, and cancer risks in check (Fig. 2). In spite of increasing evidence of the importance of miR-146a in cancer biology, there are some gaps in the research. Regulatory networks involving miR-146a in various cancers need to be explored to completely realize its mechanistic functions. Its function in immunotherapy resistance is not well explained, which restricts its therapeutic application as a target. The potential for miR-146a-based personalized treatment is also still unexplored. Yet, recent reports point to miR-146a as an emerging noninvasive biomarker for cancer detection, considering it plays a critical role in regulation of inflammatory response and immune activities in the tumor microenvironment. There is emerging evidence that the miR-146a has roles in regulation of tumor progression via the NF-κB and PI3K pathways and, therefore, has a potential connection to cancer driven by chronic inflammation. This indicates the significance of further exploration to unlock miR-146a’s maximum potential in diagnosing and treating cancer.

### miR-21

MicroRNA-21 (miR-21) is potentially carcinogenic and targets tumor suppressor proteins across nearly all cancer types [158]. miR-21 is upregulated in numerous malignancies and facilitates cell proliferation, metastasis, and treatment resistance. Targeting miR-21 with traditional chemotherapeutic drugs may augment their therapeutic efficacy and mitigate drug resistance and cancer recurrence in both in vitro and animal models [159]. miR-21 represents a viable cancer therapy target and an early cancer detection biomarker. The downregulation of tumor suppressor genes, particularly miR-21, is correlated with cancer resistance to many chemotherapeutic agents [160]. miR-21 levels were often heightened relative to surrounding normal tissue. A little overexpression of miR-21 increased tumor xenograft development and facilitated androgen-independent proliferation post-surgical castration [161]. Research into the role of miR-21 in cancer has shown various results, including one study on genetically engineered mouse models of hepatocellular carcinoma (HCC) [162]. However, the study raised concerns about using miR-21 inhibition as a therapy, whereas removing miR-21 in these models made things worse—it increased tumor growth and aggressiveness [163]. This might be because it triggered the upregulation of oncogenes and activated multiple signaling pathways out of balance. On the other hand, a different study highlighted the promise of miR-21 inhibition for treating breast cancer. Researchers using miR-21 antagomir (anti-miR-21) on 4T1 murine breast cancer cells demonstrated that tumor cells stopped multiplying as quickly and even started dying, thanks to the targeting of PTEN [164]. Even more exciting, in live animal models (VEGFR2-luc transgenic mice implanted with 4T1 cells), antagomir-21 slowed tumor angiogenesis—basically cutting off the tumor’s blood supply—by blocking the HIF-1α/VEGF/VEGFR2 pathway [165]. They confirmed this effect with bioluminescent imaging. In short, the effects of miR-21 inhibition seem to depend on the type of cancer being studied. While it is risky in liver cancer, it could offer real benefits in breast cancer (Fig. 2). Since miR-21 has a varying function in different types of cancer, its therapeutic effectiveness is very context-dependent. Cancer-specific strategies are required since, despite its well-established status as an oncogene, data such as those for hepatocellular carcinoma (HCC) suggest that its total blockage may worsen tumor growth. Targeted regulation of miR-21 or its target pathways could be a safer and more effective alternative to complete suppression. Nonetheless, clinical translation is confronted with delivery and safety issues, since off-target effects and long-term risks plague existing antagomir-based treatments. The development of tumor-specific delivery systems, such as nanoparticle-based carriers or antibody-conjugated therapies, may improve specificity and minimize side effects. Moreover, since miR-21 is involved in drug resistance, its inhibition in combination with chemotherapy or immunotherapy may enhance treatment efficacy, and combination strategies are a promising direction for future studies (Table 2).

**Table 2:** Cancer vaccines and its mechanisms

S. no	Cancer vaccine	Cancer type	Pathways involved	Mechanism of action	References
1	Sipuleucel-T (Provenge)	Prostate Cancer	Antigen Presentation Pathway, T cell Activation	Dendritic cells are exposed to a prostate cancer antigen (PAP) and GM-CSF, then reinfused to activate T cells against prostate cancer cells	[166]
2	Talimogene Laherparepvec (T-VEC)	Melanoma	HSV-1 Lytic Pathway, Antigen Presentation Pathway	Genetically modified HSV-1 virus selectively replicates in tumor cells, causing cell lysis and releasing antigens to stimulate an immune response	[167]
3	Bacillus Calmette-Guérin (BCG)	Bladder Cancer	TLR Pathway, NLR Pathway, Cytokine Pathway	BCG induces an immune response by activating toll-like receptors (TLRs) and nucleotide-binding oligomerization domain-like receptors (NLRs), leading to cytokine production and immune cell activation	[168]
4	GVAX Pancreas	Pancreatic Cancer	Antigen Presentation Pathway, T cell Activation	Pancreatic tumor cells are genetically modified to secrete GM-CSF, enhancing dendritic cell recruitment and T cell activation against pancreatic tumor antigens	[169]
5	mRNA-4157 (V940)	Multiple Cancer Types	Antigen Presentation Pathway, T cell Activation	Personalized mRNA vaccines encode neoantigens specific to the patient’s tumor, leading to antigen presentation and T cell activation	[170]
6	HPV Vaccine	Cervical Cancer, Head and Neck Cancers	Antigen Presentation Pathway, T cell Activation	HPV vaccines target HPV antigens, preventing infection and subsequent cancer development by inducing a robust immune response	[171]
7	New York-ESO-1 (NY-ESO-1) Vaccine	Various Cancer Types	Antigen Presentation Pathway, T cell Activation	The vaccine targets the NY-ESO-1 cancer/testis antigen, leading to antigen presentation and T cell-mediated immune response against tumors expressing NY-ESO-1	[172]

Additionally, miR-146a targets EGFR and MMP2, impacting cell migration, invasion, and epithelial-mesenchymal transition (EMT), essential for metastasis. miR-17-5p suppresses RUNX3, a tumor suppressor, highlighting its role in oncogenesis. Together, these miRNAs demonstrate their collective impact on cancer development by modulating shared and distinct molecular pathways, presenting opportunities for therapeutic intervention

## Conclusion and future perspective

Cancer vaccines are emerging as promising tools in oncology, utilizing the immune system to identify and destroy tumors. Ranging from tumor cell-based vaccines to innovative nucleic acid platforms, these methods show considerable potential in generating strong and lasting antitumor responses. Combining vaccines with other immunotherapeutic strategies, like immune checkpoint inhibitors and TIL or CAR-T cell therapies, enhances their effectiveness by tackling tumor diversity and immune evasion mechanisms. Moreover, miRNAs, which play crucial roles in cancer pathways, present opportunities to refine and improve vaccine-based therapies. By focusing on oncogenic and tumor suppressor miRNAs, new interventions can boost the specificity and effectiveness of cancer treatments. Despite notable progress, several obstacles still impede the broader use of cancer vaccines. Addressing challenges such as tumor-induced immunosuppression, the low immunogenicity of certain vaccines, and resistance mechanisms is essential. Moreover, regulatory and logistical hurdles greatly affect the clinical application of cancer vaccines. Strict approval procedures, the costliness of development, and standardization requirements in manufacturing and distribution make sweeping adoption difficult. Personalized vaccine approaches need careful biomarker identification and patient-specific formulation, making clinical translation more complicated. Future studies should aim to integrate multi-omics approaches, including proteomics and genomics, to discover new biomarkers for personalized vaccine development. The combination of miRNA modulation with cancer vaccines is an exciting area of exploration, with the potential to enhance immune responses and overcome treatment resistance. Additionally, advancements in delivery systems, particularly nanotechnology-based ones, could enhance vaccine stability and targeting. A collaborative effort across various fields, alongside regulatory streamlining and improved infrastructure, will be crucial to unlocking the full potential of vaccines for different cancer types, ultimately leading to better patient outcomes.

## Data Availability

No datasets were generated or analyzed during the current study.
